# Overexpression of Chromatin Remodeling Factor SRG3 Down-Regulates IL1β-Expressing M1 Macrophages and IL17-Producing T Cells in Adipose Tissues

**DOI:** 10.3390/ijms252111681

**Published:** 2024-10-30

**Authors:** Jungmin Jeon, Sung Won Lee, Hyun Jung Park, Yun Hoo Park, Tae-Cheol Kim, Sujin Lee, Seyeong Lee, Luc Van Kaer, Seokmann Hong

**Affiliations:** 1 Department of Integrative Bioscience and Biotechnology, Institute of Anticancer Medicine Development, Sejong University, Seoul 05006, Republic of Korea; jjm4165@gmail.com (J.J.); 0402parkhj@gmail.com (H.J.P.); dbsgn703@gmail.com (Y.H.P.); mitalus1@gmail.com (T.-C.K.); lsj1262487@gmail.com (S.L.); lsyjhs0218@gmail.com (S.L.); 2 Department of Biomedical Laboratory Science, College of Health and Biomedical Services, Sangji University, Wonju 26339, Republic of Korea; sungwonlee@sangji.ac.kr; 3 Department of Pathology, Microbiology and Immunology, Vanderbilt University School of Medicine, Nashville, TN 37232, USA; luc.van.kaer@vumc.org

**Keywords:** SWItch3-related gene (SRG3), adipose tissue, M1 macrophage, IL17, glucose tolerance

## Abstract

The SWItch3-related gene (SRG3) is a core component of ATP-dependent SWI/SNF complexes, which are crucial for regulating immune cell development and function (e.g., macrophages and CD4^+^ T cells), embryonic development, and non-immune cell differentiation. Notably, SRG3 overexpression has been shown to polarize macrophages in the central nervous system toward an anti-inflammatory M2 phenotype, thereby protecting against the development of experimental autoimmune encephalomyelitis in mice. However, the effect of SRG3 on immune responses in adipose tissues remains unclear. To address this issue, we examined the cellularity and inflammatory status of adipose tissue in B10.PL mice overexpressing the SRG3 gene under the ubiquitous β-actin promoter (SRG3^β-actin^). Interestingly, SRG3 overexpression significantly reduced adipocyte size in both white and brown adipose tissues, without affecting the overall adipose tissue weight. Such phenotypic effects might be associated with the improved glucose tolerance observed in SRG3^β-actin^ B10.PL mice. Moreover, we found that SRG3 overexpression down-regulates IL1β-expressing M1 macrophages, leading to a significant decrease in the M1/M2 macrophage ratio. Additionally, SRG3^β-actin^ B10.PL mice showed a dramatic reduction in neutrophils as well as IL1β- and IL17-producing T cells in adipose tissues. Taken together, our results indicate that SRG3 plays a vital role in maintaining immune homeostasis within adipose tissues.

## 1. Introduction

The SWItch3-related gene (SRG3) is a core subunit of various ATP-dependent SWI/SNF complexes. The SRG3 protein contains a leucine-zipper motif that stabilizes the SWI/SNF complex, and a proline- and glutamine-rich region that facilitates the opening or closing of chromatin structures—thereby regulating gene expression in response to various cellular stimuli [1]. Although it has been reported that SRG3 plays an essential role in embryonic and brain development [2], it is also crucial for regulating the differentiation of immune cells (i.e., macrophages, CD4^+^ T cells, and B cells) [3,4,5]. Moreover, SRG3 overexpression confers protective effects on lipopolysaccharide (LPS)/D-galactosamine (D-GalN)-induced sepsis, which correlated with the phenotypic conversion of M1 to M2 macrophages in C57BL/6 (B6) mice [5]. B10.PL mice dually transgenic (Tg) for beta-actin promoter-driven SRG3 (SRG3^β-actin^ Tg) and a myelin basic protein (MBP)-specific T cell receptor (TCR) are partially protected against experimental autoimmune encephalomyelitis (EAE) compared with MBP TCR Tg B10.PL mice [6]. Under steady state conditions, insulin stimulation increases SRG3 expression to remodel chromatin and open the ADD1/SREBP1c promoter, by which SRG3 regulates insulin-dependent gene expression in adipocytes. Consequently, SRG3-overexpressing B6 mice exhibit an improved glucose tolerance, suggesting that SWI/SNF chromatin remodeling complexes contribute to regulating metabolic disorders [7]. However, the effect of SRG3 on immune responses in the adipose tissue remains largely unknown.

Blood glucose is the primary energy source for tissues such as the brain. Glucagon and insulin are two essential hormones that control blood glucose levels in opposing ways: glucagon increases the release of glucose into the bloodstream through glycogenolysis, whereas insulin promotes glucose uptake into muscle and adipose tissues through glycogenesis [8]. Glucose intolerance refers to the body’s inability to absorb exogenous glucose from diets, resulting in abnormally high blood glucose levels, which represents an intermediate stage in the progression toward hyperglycemia (also known as the prediabetes stage of type 2 diabetes) [9]. Hyperglycemia induces increased reactive oxygen species production in adipocytes, leading to a robust inflammatory response [10]. In obesity, M1 macrophages polarized by pro-inflammatory mediators such as TNFα secrete inflammatory molecules (e.g., cytokines and chemokines) in white adipose tissue (WAT), which can contribute to the development of insulin resistance. In addition, IL4-induced M2 macrophages produce IL10 to promote insulin sensitivity within WAT and secrete catecholamine to induce beige fat formation which facilitates thermogenesis [11]. The transplantation of brown adipose tissues (BAT) attenuates glucose intolerance, and insulin resistance increases following myocardial infarction (MI) injury by inhibiting inflammatory gene expression in the WAT and liver [12].

The neutrophil-to-lymphocyte ratio, a significant predictor of glucose intolerance in adult subjects with severe obesity, may serve as an early diagnostic marker of glucose intolerance [13]. Neutrophil depletion has been shown to effectively suppress an increase in fasting blood glucose levels under high-fat diet conditions, underscoring their crucial role in glucose homeostasis [14]. It has been reported that competitive binding of an IL1 receptor (IL1R) antagonist with the IL1R diminishes basal-fed plasma glucose levels in type 2 diabetic Goto-Kakizaki rats [15]. Moreover, IL17 treatment hinders glucose uptake in 3T3-L1 adipocytes in vitro, and young IL17-deficient mice display increased glucose tolerance and insulin sensitivity, indicating that the inflammatory cytokine IL17 acts as a negative regulator of glucose metabolism in vivo [16]. Given the incomplete understanding of the immunoregulatory role of chromatin remodeling factor SRG3 in the adipose tissue, we hypothesized that SRG3 expression facilitates immune homeostasis in adipose tissues. To address this issue, we investigated the effect of SRG3 overexpression in mice on the immune status in white and brown adipose tissues, with the ultimate goal to guide future research directions related to clinical applications.

## 2. Results

### 2.1. SRG3 Overexpression Improves Glucose Tolerance, Which Is Associated with a Reduction in Adipocyte Size in Adipose Tissues

**Figure 1:**
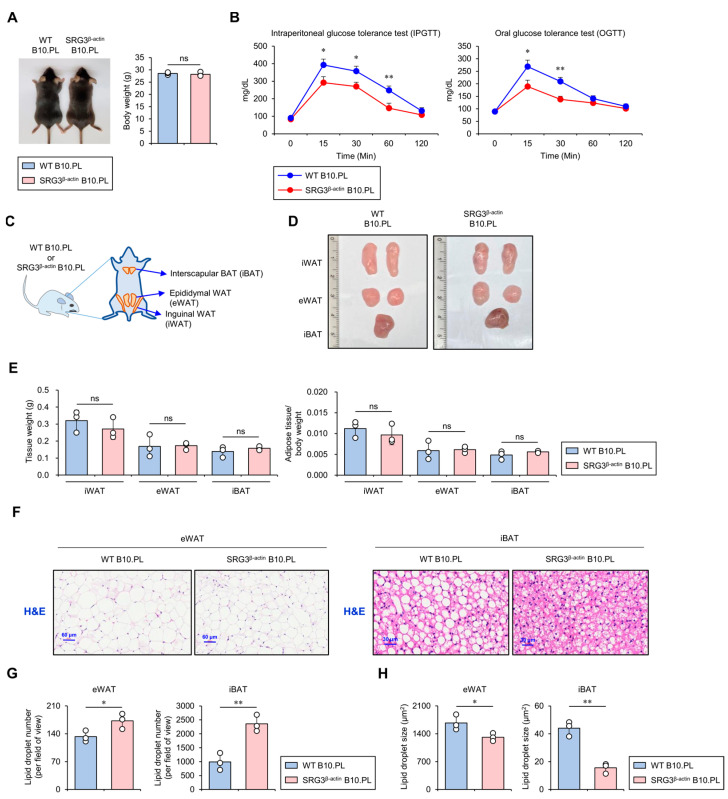
SRG3 overexpression improves glucose tolerance, which is associated with reduced adipocyte size in adipose tissues. (**A**) The body weight was measured in WT B10.PL and SRG3^β-actin^ B10.PL mice. (**B**) Mice were analyzed for oral or intraperitoneal glucose tolerance tests at the indicated time points. (**C**) Schematic diagram showing the localization of mouse adipose tissues, including iWAT, eWAT, and iBAT. (**D**) Representative pictures of the iWAT, eWAT, and iBAT were obtained in WT B10.PL and SRG3^β-actin^ B10.PL mice. (**E**) Adipose tissue weight and adipose tissue/body weight ratio of iWAT, eWAT, and iBAT were measured in WT B10.PL and SRG3^β-actin^ B10.PL mice. (**F**) eWAT and iBAT were sectioned and stained with H&E. (**G**,**H**) The number (**G**) and size (**H**) of adipocyte lipid droplets in eWAT and iBAT were quantified by ImageJ software (v1.54j). The mean values ± SD (*n* = 3 in (**A**–**H**); per group in the experiment; one-way ANOVA; * *p* < 0.05 and ** *p* < 0.01) are shown. One representative experiment of two experiments is shown. ns, not significant.

Previous studies have reported that SRG3 Tg mice on the B6 genetic background display improved glucose tolerance and increased insulin sensitivity in their adipose tissues [7]. To address whether such effects of SRG3 overexpression on glucose tolerance apply to mice of the B10.PL genetic background used in this study, we injected glucose orally or intraperitoneally (i.p.) into wild-type (WT) and SRG3^β-actin^ B10.PL mice after 24 h of fasting and performed a glucose tolerance test (GTT) over 120 min. At 15, 30, and 60 min after the oral glucose injection, we found that the blood glucose levels were significantly lower in SRG3^β-actin^ B10.PL mice than in WT control mice (Figure 1A,B). These results strongly indicate that ubiquitous overexpression of SRG3 confers improved glucose tolerance to SRG3^β-actin^ B10.PL mice.

Large adipocytes release pro-inflammatory cytokines, which can promote glucose intolerance, insulin resistance, and type 2 diabetes, whereas small adipocytes tend to be associated with improved glucose regulation and increased insulin sensitivity [17]. Based on these previous findings, we investigated whether SRG3 overexpression can affect the quantity and quality of adipocytes (e.g., cell number and size distribution). We analyzed the weight of adipose tissues (e.g., inguinal WAT [iWAT], epididymal WAT [eWAT], and interscapular BAT [iBAT]) in WT and SRG3^β-actin^ B10.PL mice. Although we were unable to identify significant differences in adipose tissue weight in these animals (Figure 1C–E), intriguingly, our data showed a smaller average adipocyte diameter in the eWAT and iBAT of SRG3^β-actin^ B10.PL mice compared to WT B10.PL mice (Figure 1F–H). Overall, these results indicate that overexpression of SRG3 might be associated with inhibiting fat storage in adipocytes.

### 2.2. SRG3 Overexpression Induces Selective Down-Regulation of the M1 Macrophage Population in the Adipose Tissues of SRG3^β-actin^ B10.PL Mice

**Figure 2:**
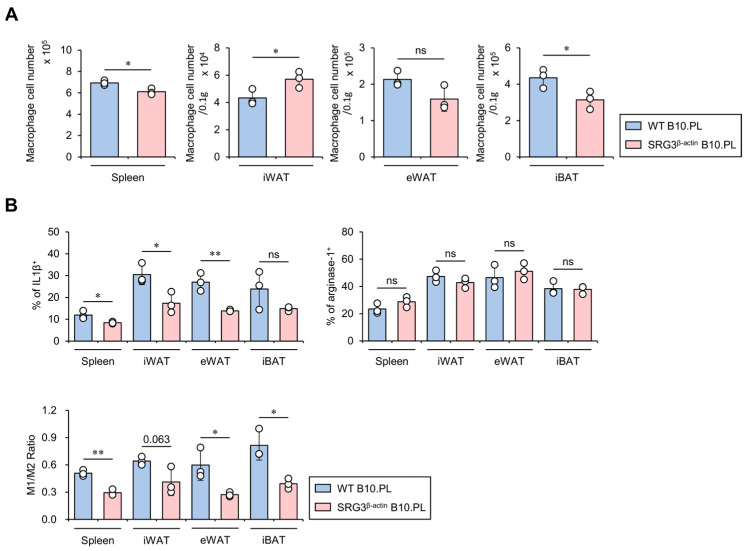
SRG3 overexpression induces selective down-regulation of the M1 macrophage population in the adipose tissues of SRG3^β-actin^ B10.PL mice. (**A**) The cell number of splenic, iWAT, eWAT, and iBAT macrophages (CD45^+^F4/80^+^CD11b^+^) was analyzed by flow cytometry in WT B10.PL and SRG3^β-actin^ B10.PL mice. (**B**) The frequency of IL1β^+^ or arginase-1^+^ macrophages and the M1/M2 ratio in the spleen, iWAT, eWAT, and iBAT were determined by flow cytometry. The mean values ± SD (*n* = 3 in (**A**,**B**); per group in the experiment; one-way ANOVA; * *p* < 0.05 and ** *p* < 0.01) are shown. One representative experiment of two experiments is shown. ns, not significant.

We have previously shown that ubiquitous overexpression of SRG3 protects B10.PL and B6 mice against EAE and septic shock, respectively, via up-regulation of M2 macrophages [6]. It has been reported that, under lean conditions, IL4-induced M2 macrophages secrete anti-inflammatory cytokines in adipose tissues, leading to increased glucose uptake. However, under obese conditions, IFNγ-polarized M1 macrophages produce large amounts of pro-inflammatory cytokines in adipose tissues, consequently promoting insulin resistance [18]. Therefore, we investigated the impact of ubiquitous SRG3 overexpression on the size and M1/M2 polarization status of the macrophage population in adipose tissues. For this purpose, we isolated mononuclear cells from adipose tissues of WT and SRG3^β-actin^ B10.PL mice and assessed them for M1 and M2 macrophage polarization using flow cytometry. We found that the number of macrophages was slightly but significantly reduced in the spleen and iBAT of SRG3^β-actin^ B10.PL mice compared to WT B10.PL mice, whereas the number of these cells was moderately but significantly increased in iWAT of SRG3^β-actin^ B10.PL mice compared to WT B10.PL mice (Figure 2A). In addition, SRG3^β-actin^ B10.PL mice showed an increased frequency of macrophages producing IL1β but not arginase-1 in the spleen, iWAT, and eWAT compared with WT B10.PL mice, indicating that SRG3 overexpression selectively decreases M1 macrophages. Therefore, the M1 (IL1β)/M2 (arginase-1) ratio among macrophages was significantly decreased in the spleen and eWAT but tended to be decreased in the iWAT of SRG3^β-actin^ B10.PL mice compared with WT B10.PL mice (Figure 2B). These results indicate that improved glucose tolerance due to SRG3 overexpression might be associated with a reduction in the M1/M2 ratio in adipose tissues.

### 2.3. Correlation Analysis of SRG3 and Macrophage Subset Gene Expression in Human Adipose Tissues

**Figure 3:**
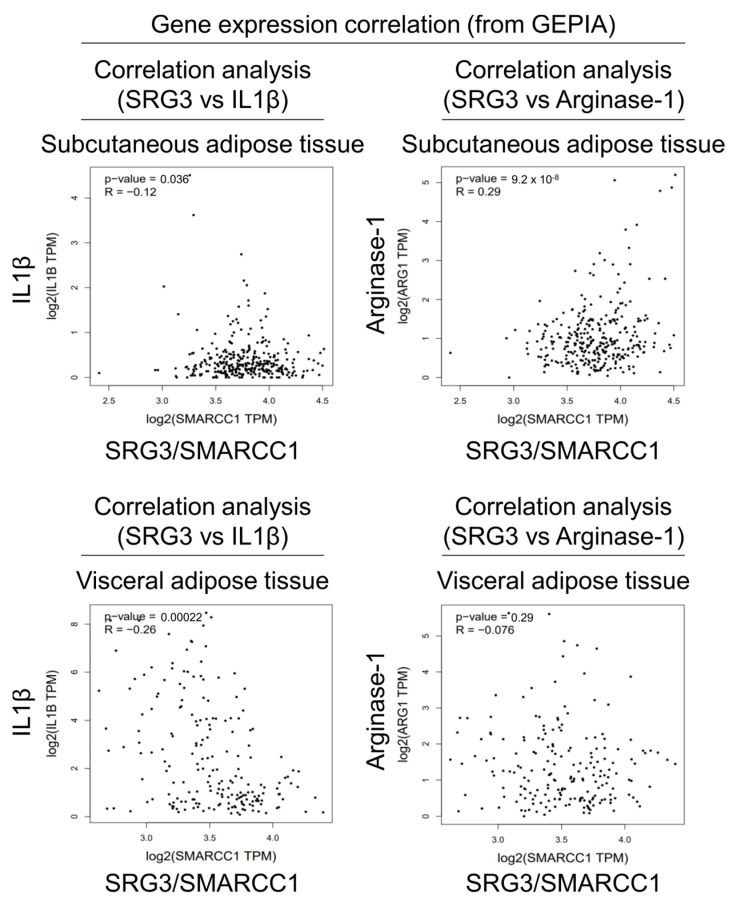
Correlation analyses of SRG3 and macrophage subset gene expression in human adipose tissues. Pearson correlation analysis of *SRG3* and *IL1B* or *ARG1* gene expression was conducted using the human subcutaneous and visceral adipose tissue data from the GEPIA (http://gepia.cancer-pku.cn/index.html, accessed on 27 February 2024) tool (TPM; transcripts per million reads).

Since our results suggested that SRG3 plays a critical role in determining the polarization of adipose tissue macrophages in mice, we decided to examine whether there is any correlation between SRG3 gene (*SMARCC1)* expression and M1/M2-related marker gene expression profiles (*IL1B* and *ARG1*) in human adipose tissues. To address this issue, we analyzed data from the Gene Expression Profiling Interactive Analysis (GEPIA) website, which includes expression data for human subcutaneous (iWAT) and visceral (eWAT) adipose tissues. Our analysis revealed that *SMARCC1* expression negatively correlates with *IL1B* expression in iWAT (R = −0.12, *p* = 0.036) and eWAT (R = −0.26, *p* = 0.00022) (Figure 3). Additionally, *SMARCC1* expression positively correlated with *ARG1* expression in iWAT (R = 0.29, *p* = 9.2 × 10^−8^) but not in eWAT (R = −0.076, *p* = 0.29) (Figure 3). Thus, this correlation between the expression of the SRG3 gene and M1/M2-marker genes in human adipose tissues supports our findings with adipose tissues from SRG3 overexpressing mice.

### 2.4. SRG3 Overexpression Down-Regulates the Accumulation of IL1β- and IL17-Producing T Cells in Adipose Tissues

**Figure 4:**
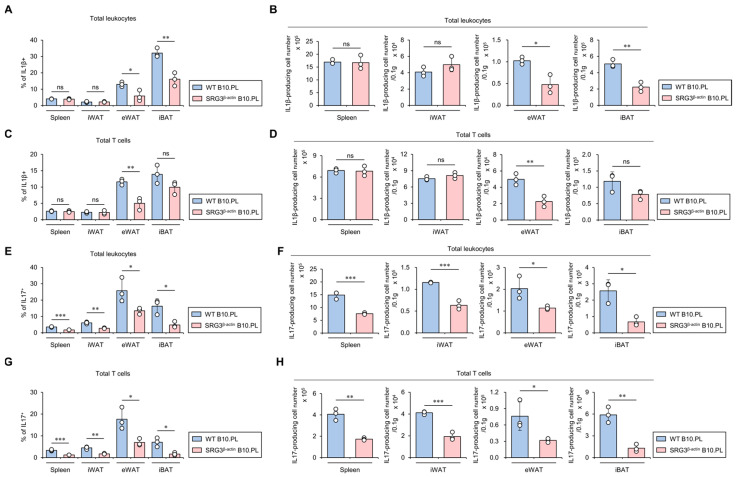
SRG3 overexpression down-regulates the accumulation of IL1β- and IL17-producing T cells in adipose tissues. (**A**–**D**) The frequency (**A**,**C**) and cell number (**B**,**D**) of IL1β^+^ leukocytes (CD45^+^) and IL1β^+^ T cells (CD45^+^CD3^+^) from the spleen, iWAT, eWAT, and iBAT were assessed by flow cytometry in WT B10.PL and SRG3^β-actin^ B10.PL mice. (**E**–**H**) The frequency (**E**,**G**) and cell number (**F**,**H**) of IL17^+^ leukocytes (CD45^+^) and IL17^+^ T cells (CD45^+^CD3^+^) from the spleen, iWAT, eWAT, and iBAT were determined by flow cytometry in WT B10.PL and SRG3^β-actin^ B10.PL mice. The mean values ± SD (*n* = 3 in (**A**–**H**); per group in the experiment; one-way ANOVA; * *p* < 0.05, ** *p* < 0.01, and *** *p* < 0.001) are shown. One representative experiment of two experiments is shown. ns, not significant.

IL1β contributes to the development of impaired glucose tolerance in mice [19]. Therefore, we investigated whether SRG3 overexpression down-regulates IL1β production in adipose tissue immune cells. As expected, we found that SRG3 overexpression significantly inhibits the total cell number of IL1β-producing immune cells in eWAT and iBAT. Intriguingly, IL1β-producing T cells were profoundly reduced only in the eWAT of SRG3^β-actin^ B10.PL mice compared to WT B10.PL mice (Figure 4A–D). Since anti-IL17A monoclonal antibodies inhibit serum glucose levels in both mice and humans, ultimately improving hyperglycemia [20], we examined whether SRG3 overexpression modulates IL17-producing immune cells in the adipose tissues of B10.PL mice. We found that the frequency and number of IL17-producing T cells in the spleen and adipose tissues were significantly lower in SRG3^β-actin^ B10.PL mice than WT B10.PL mice (Figure 4E–H). Moreover, similar to our results for conventional αβ T cells, IL17-producing γδ T cells were significantly lower in iWAT and eWAT from SRG3^β-actin^ B10.PL mice than WT B10.PL mice (Figure S1). Collectively, these results strongly indicate that the reduction in adipocyte size and increased glucose tolerance due to SRG3 overexpression are associated with down-regulated IL1β and IL17 production in adipose tissue immune cells.

### 2.5. SRG3 Overexpression Limits the Accumulation of Neutrophils but Not Eosinophils in Adipose Tissues

**Figure 5:**
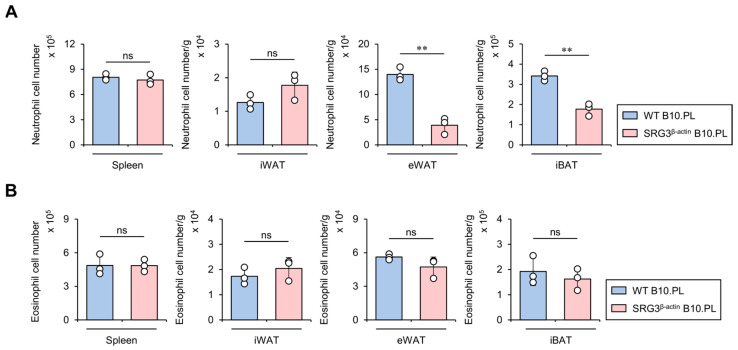
SRG3 overexpression limits the accumulation of neutrophils but not eosinophils in adipose tissues. (**A**) The cell number of neutrophils (CD45^+^Gr-1^+^CD11b^+^) from the spleen, iWAT, eWAT, and iBAT was measured by flow cytometry in WT B10.PL and SRG3^β-actin^ B10.PL mice. (**B**) The cell number of eosinophils (CD45^+^Siglec-F^+^CD11b^+^) from the spleen, iWAT, eWAT, and iBAT was assessed by flow cytometry in WT B10.PL and SRG3^β-actin^ B10.PL mice. The mean values ± SD (*n* = 3 in (**A**,**B**); per group in the experiment; one-way ANOVA; ** *p* < 0.01) are shown. One representative experiment of two experiments is shown. ns, not significant.

It has been reported that neutrophils infiltrate into eWAT in high-fat diet (HFD)-fed mice, ultimately hindering glucose tolerance and insulin sensitivity [21]. Thus, we examined whether SRG3 overexpression influences the accumulation of neutrophils in adipose tissues from B10.PL mice. To address this issue, we assessed the neutrophil cell numbers in the spleen and adipose tissues (e.g., iWAT, eWAT, and iBAT). We found that SRG3 overexpression reduces the number of adipose tissue neutrophils, and these effects appeared to be restricted to eWAT and iBAT, as they were not detected in the spleen and iWAT (Figure 5A). Previous studies have reported that eosinophil-deficient mice display significantly elevated glucose levels in GTT and higher HFD-induced adipose tissue inflammation, suggesting that eosinophils are regulators of glucose homeostasis [22]. Thus, we further investigated whether SRG3 overexpression impacts the eosinophil population in the adipose tissues of B10.PL mice. Unexpectedly, SRG3 overexpression did not alter the number of adipose tissue eosinophils in B10.PL mice (Figure 5B). These results support the notion that the improved glucose tolerance mediated by SRG3 overexpression might be attributed in part to down-regulation of the neutrophil population in adipose tissues under steady-state conditions.

## 3. Discussion

Here, we demonstrated that SRG3^β-actin^ B10.PL mice display an enhanced glucose tolerance, which was associated with the down-regulation of pro-inflammatory macrophages (i.e., decreased M1/M2 ratio). In addition, SRG3 overexpression reduced the prevalence of pro-inflammatory immune cells (e.g., neutrophils and IL1β- and IL17-producing T cells) in adipose tissues.

Whole-body knockout (KO) of the SRG3 gene exhibits early lethality in mice, which is associated with significant abnormalities in early embryogenesis and brain development [1]. One study demonstrated that the overexpression of SRG3 in the T-cell lineage using the human CD2 promoter exacerbates the development of myelin oligodendrocyte glycoprotein (MOG)-induced EAE in B6 mice [23]. Consistent with this study, we previously reported that SRG3 overexpression driven by the CD2 promoter accelerates the progression of MBP-induced EAE in MBP-specific TCR Tg B10.PL mice [6]. Such pro-inflammatory effects of T cell-specific SRG3 expression are supported by a previous study showing that SRG3 is required to generate Th17 cells in an RORγt-dependent manner [4]. In contrast, β-actin promoter-driven SRG3 overexpression attenuated the severity of MBP-induced EAE and reduced the clinical symptoms of LPS/D-GalN-induced sepsis via alternative macrophage activation [5,6]. These previous studies suggest that, depending on the cell type studied (e.g., myeloid cells vs. lymphoid cells), SRG3 overexpression confers distinct outcomes in inflammatory diseases. Thus, cell-specific fine-tuning of SRG3 expression in the adipose tissue might be applicable to controlling metabolic diseases.

It is well known that IL1β and IL23 can activate γδ T cells to secrete IL17 even in the absence of TCR stimulation [24]. γδ T cell-derived IL17 plays a critical role during the early stage of EAE induction [25]. Moreover, previous studies have provided evidence that neutrophil-derived IL1β is required for IL17 production by γδ T cells in the injured central nervous system during EAE pathogenesis and, conversely, γδ T cell-derived IL17 drives the recruitment of IL1β-producing neutrophils [25]. Based on previous reports showing that IL17 and IL1β both worsen glucose intolerance [16,19], together with our findings that IL17-producing γδ T cells and IL1β-producing neutrophils are dramatically reduced in the adipose tissues of SRG3^β-actin^ B10.PL mice, it will be interesting to investigate how crosstalk between γδ T cells and neutrophils (i.e., γδ T cell-neutrophil axis) contributes to regulating immune responses in adipose tissues.

It has been reported that skeletal muscle takes up about 70–80% of all blood glucose during insulin stimulation, whereas adipose tissue absorbs approximately 10–20% of the remaining blood glucose, highlighting skeletal muscle as a major site of glucose homeostasis [26]. Interestingly, the mammalian SWI/SNF components Brahma (BRM) and BRG1 (BRM-related gene 1), which generate complexes with SRG3, induce myogenic differentiation factor (MyoD)-facilitated muscle differentiation [27]. Moreover, Brm and Brg1 promote the expression of endogenous smooth muscle-specific genes stimulated by myocardin in SW13 adrenocortical cell lines [28]. Based on these reports, we cannot exclude the possibility that the positive effects of SRG3 overexpression on glucose regulation we observed might depend in large part on skeletal muscle. Thus, in future studies it will be exciting to explore the potential role of SRG3 on glucose metabolism in skeletal muscle.

Mitochondrial matrix protein Letmd1 KO mice generated via CRISPR-Cas9 gene editing exhibit decreased expression levels of thermogenic markers such as UCP1 and EBF2 in BAT, leading to impaired thermogenesis during cold exposure. Moreover, Brg1 is essential for Letmd1-dependent thermogenic gene regulation following cold exposure [29]. Conditional KO of the SMARCB1 gene encoding a subunit of the SWI/SNF complex in preadipocytes exhibited reduced expression of BAT-specific genes such as *Prdm16* and *Ucp1* in BAT [30]. Consistent with these previous studies, we noted that BAT from SRG3^β-actin^ B10.PL mice contains multilocular adipocytes that are colored more deeply pink and are smaller than the adipocytes of WT B10.PL mice (Figure 1F). Thus, it will be interesting to investigate whether SRG3 overexpression in BAT can enhance thermogenesis.

It has been previously reported that SRG3 is necessary for insulin-dependent ADD1/SREBP1c expression in 3T3-L1 adipocytes, and insulin treatment enhanced SRG3 expression in these cells [7]. In addition, SRG3 Tg B6 mice showed improved glucose regulation under steady state conditions [7]. Moreover, we analyzed SRG3/SMARCC1 expression in various primary cells from healthy human visceral adipose tissues using the human protein atlas website. We found that the SRG3 gene is highly expressed in adipose progenitor cells (Figure S2), suggesting the potential role of SRG3 in adipocyte homeostasis, since adipose progenitors are involved in maintaining fat homeostasis and insulin sensitivity [31]. Previous studies have shown that certain adipokines, such as omentin-1, suppress adipose tissue inflammation in high-fat diet-induced obese mice [32]. Furthermore, omentin-1 significantly inhibited M1 macrophage polarization in the LPS-induced in vitro inflammatory periodontitis model [33]. It has been reported that omentin-1, an adipose tissue-derived adipokine, plays crucial roles in maintaining glucose homeostasis and insulin sensitivity and exerting anti-inflammatory functions [34]. Thus, we performed correlation analysis using data from the GEPIA website, which includes expression data for human iWAT and eWAT. Our analysis revealed that SRG3/SMARCC1 expression positively correlates with omentin-1 (encoded by the *ITLN1* gene) expression in eWAT (R = 0.28, *p* = 6.6 × 10^−5^) but not iWAT (R = −0.08, *p* = 0.15) (Figure S3). These data suggest that SRG3 expression in adipose tissues influences the expression of adipose tissue-derived anti-inflammatory factors, such as omentin-1, which might contribute to down-regulating IL1β in M1 macrophages and IL17 in T cells within the adipose tissue. Thus, in future studies, it will be interesting to investigate whether siRNA-mediated knockdown of SRG3 in visceral adipocytes can down-regulate omentin-1 expression, consequently leading to anti-inflammatory effects on macrophages and T cells.

In conclusion, our results show that ubiquitous overexpression of SRG3 may modulate the immune homeostasis of adipose tissues by reducing pro-inflammatory M1 macrophages and IL17-producing T cell populations in both WAT and BAT, which correlates with regulated glucose metabolism (i.e., improved glucose tolerance). According to previous studies, the expression of BAF60a, an SWI/SNF complex subunit, is reduced in the stromal vascular fraction of eWAT in db/db mice [35]. Similarly, other studies have shown that in conditions of diabetes and obesity, the expression of SMARCA5, a member of the SWI/SNF family of proteins, is reduced in endothelial cells of the lungs and heart [36]. Moreover, in streptozotocin-induced diabetes models, SRG3 expression is decreased in eWAT, which can be reversed by insulin treatment [7]. However, further research is warranted to fully understand the contribution of SRG3 overexpression to balancing between immune and metabolic homeostasis in adipose tissues and its potential relevance to metabolic diseases such as obesity and type 2 diabetes.

## 4. Materials and Methods

### 4.1. Study Design

This study was designed to determine the effect of SRG3 overexpression on immune responses in adipose tissue. To address this issue, we established SRG3^β-actin^ B10.PL mice, which overexpress SRG3 ubiquitously. Immune cells were harvested from white and brown adipose tissues of SRG3^β-actin^ B10.PL mice and further analyzed by flow cytometry.

### 4.2. Mice

SRG3^β-actin^ B6 mice were provided by Dr. Rho H. Seong (Seoul National University, Seoul, Republic of Korea), and SRG3^β-actin^ B10.PL mice were generated by backcrossing SRG3^β-actin^ B6 mice onto the B10.PL background for more than ten generations. All mice used in this study were male mice on a B10.PL genetic background, were maintained at Sejong University, and were used for experiments at 6–12 weeks of age. These mice were maintained on a 12 h light/12 h dark cycle in a temperature-controlled barrier facility with free access to food and water. They were fed a γ-irradiated sterile diet (Purina 38057 [moisture (8.10%), crude protein (21.60%), crude fat (6.97%), crude fiber (4.00%), crude ash (6.06%), calcium (1.08%), and phosphorus (0.67%)], Cargill Agri Purina Inc., Seongnam-si, Republic of Korea) and provided with autoclaved tap water. According to the manufacturer’s description, the levels of phytoestrogens are sufficiently low so that they do not significantly influence the experimental outcomes [37]. Age- and sex-matched mice were used for all experiments. The animal experiments were approved by the Institutional Animal Care and Use Committee at Sejong University (SJ-20161102,15 November 2016).

### 4.3. Genotyping of Mice

To verify the transgene’s integration, genomic DNA from tail biopsies was used to amplify an 800 bp fragment that was only detectable in SRG3^β-actin^ mice carrying the SRG3 transgene. The following primers were used for genotyping SRG3^β-actin^ mice by PCR: forward 5′-GAC TAG ACC AAA CAT CTA CCT C-3′; reverse 5′-GTC AAC TGA GCG ACT GGA TC-3′. The protocol used in this study is the same as our previous study [6].

### 4.4. Glucose Tolerance Test (GTT)

For the oral and intraperitoneal GTT, mice were fasted for 12 h and subsequently treated with oral gavage or intraperitoneal injection of glucose (2 g glucose/kg of body weight). Blood samples were collected from the tail vein at 0 (before glucose administration), 15, 30, 60, and 120 min after glucose administration. After obtaining all the samples, blood glucose levels were measured using a glucometer (Accu-Chek, Roche Diagnostics, São Paulo, SP, Brazil).

### 4.5. Hematoxylin and Eosin (H&E) Staining and Analysis of Adipose Tissues

Adipose tissues, including iWAT, eWAT, and iBAT, were fixed in 4% paraformaldehyde, embedded in paraffin, and sectioned into 6 µm-thick slices using a microtome (RM 2235, Leica, Wetzlar, Germany). The sections were stained with H&E for histological analysis. Whole-slide digital images were acquired with the Korea Non-clinical Technology Solution Center (Seongnam-si, Republic of Korea). The scanned H&E-stained images were analyzed using Motic Digital Slide Assistant software (version 1.0.7.60, Hong Kong). Analyses of lipid droplet sizes and numbers were quantified using ImageJ software (v1.54j). A total of 10 different areas per H&E-stained image were analyzed, and a representative image is shown.

### 4.6. Isolation of Immune Cells from Adipose Tissues

For single-cell preparation, iWAT, eWAT, and iBAT were harvested separately. Subsequently, these adipose tissues were finely minced and digested in 1 mg/mL collagenase IV (Sigma, St. Louis, MO, USA) in RPMI, and digestion was performed at 37 °C with thorough shaking for 1 h. The harvested cells were filtered through a 70 µm nylon cell strainer (BD Falcon, Franklin Lakes, NJ, USA) and washed twice with PBS (2000 rpm, 10 min, 4 °C) for single-cell suspensions. After centrifugation, the floating adipocytes were aspirated, and the stromal vascular fraction (SVF) pellet was resuspended in ammonium-chloride-potassium (ACK) lysis buffer for 2–3 min and washed with PBS. Isolated SVF cells were harvested for flow cytometry analysis.

### 4.7. Flow Cytometry

The following monoclonal antibodies (mAbs) were obtained from BD Biosciences (San Jose, CA, USA): Fluorescein isothiocyanate (FITC)-conjugated anti-CD3ε (clone 145-2C11); phycoerythrin (PE)-conjugated anti-Siglec-F (clone E50-2440); PE- or PE/Cy7-conjugated anti-CD11b (clone M1/70). The following mAbs from Thermo Fisher Scientific (Waltham, MA, USA) were used: FITC-conjugated anti-CD19 (clone ID3); PE-conjugated anti-IL1β (clone NJTEN3); PE-conjugated anti-IL17A (clone eBio17B7); PE- or allophycocyanin (APC)-conjugated anti-Ly-6G (Gr-1) (clone 1A8-Ly6g); PE/Cy7-conjugated anti-γδ TCR (clone GL3); FITC- or APC-conjugated anti-F4/80 (clone BM8). The following mAbs from BioLegend (San Diego, CA, USA) were used: FITC- or APC-conjugated anti-CD45 (clone 30-F11); APC-conjugated anti-CD3ε (clone 17A2). The following mAb from R&D systems (Minneapolis, MN, USA) was used: PE-conjugated anti-Arginase-1. To perform surface staining, cells were harvested from the spleen, iWAT, eWAT, and iBAT separately and washed twice with cold 0.5% BSA-containing PBS (FACS buffer). The cells were incubated with anti-CD16/CD32 mAbs (clone 2.4G2) on ice for 10 min to block the Fc receptor and stained with fluorescently labeled mAbs. Flow cytometric data were acquired using a FACSCalibur flow cytometer (Becton Dickinson, San Jose, CA, USA) and analyzed using FlowJo software (version 8.7; Tree Star, Ashland, OR, USA).

### 4.8. Intracellular Cytokine Staining

To perform intracellular staining, single-cell suspensions from each tissue were incubated with 10 μg/mL intracellular protein transport inhibitor (brefeldin A) in RPMI medium (Gibco BRL, Gaithersburg, MD, USA) for 2 h at 37 °C. The cells were stained for cell-specific surface markers, then fixed with 1% paraformaldehyde in PBS, washed with cold (4 °C) FACS buffer, and permeabilized with 0.5% saponin in PBS for 10 min. Subsequently, the permeabilized cells were stained for 30 min at RT with the indicated anti-cytokine mAbs (PE-conjugated anti-IL1β, anti-Arginase-1, anti-IL17A, PE-conjugated rat IgG isotype control mAbs). Samples (more than 5000 cells) were acquired using a BD FACSCalibur, and the data were analyzed using the FlowJo software package (version 8.7; Tree Star, Ashland, OR, USA) [38].

### 4.9. Data Collection in the GEPIA

Correlation analysis between SRG3 (*SMARCC1*) and M1/M2-related genes (*IL1B* and *ARG1*)/Omentin-1 (*ITLN1*) was conducted using RNA-seq data of healthy human subcutaneous and visceral adipose tissue from a bioinformatics GEPIA database [39]. The data utilized in this study are credited to the GEPIA database. Data summary images were obtained from http://gepia.cancer-pku.cn/index.html (accessed on 27 February 2024).

### 4.10. Data Collection in the Human Protein Atlas

RNA-seq data of SRG3/SMARCC1 mRNA expression in healthy human liver and lung tissues were re-organized by the tissue atlas program using data generated by the Human Protein Atlas [40]. Data credit: Human Protein Atlas. Data summary images were obtained from proteinatlas.org via https://www.proteinatlas.org/ENSG00000173473-SMARCC1/celltype (accessed on 17 October 2024).

### 4.11. Statistical Analysis

Graphs were drawn using Microsoft Excel 2019 (Microsoft, Redmond, WA, USA). One-way ANOVA analysis was carried out using VassarStats (http://vassarstats.net/anova1u.html) (accessed on 21 August 2024). * *p* < 0.05, ** *p* < 0.01, and *** *p* < 0.001 were considered significant in the one-way ANOVA. 

## Data Availability

The data will be available from the corresponding author upon reasonable request.

## Supplementary Materials

The supporting information can be downloaded at https://www.mdpi.com/article/10.3390/ijms252111681/s1.
