# Supplementary material for: Overexpression of Chromatin Remodeling Factor SRG3 Down-Regulates IL1β-Expressing M1 Macrophages and IL17-Producing T Cells in Adipose Tissues
Source: Int J Mol Sci. 2024 Oct 30;25(21):11681. doi: 10.3390/ijms252111681 (PMC11546064; doi:10.3390/ijms252111681)
Supplement: Supplementary file 1 [file ijms-25-11681-s001.zip › ijms-3274407-supplementary.pdf]

Supplementary Materials to:

Overexpression of Chromatin Remodeling Factor SRG3 Down-regulates IL1β-Expressing M1 Macrophages and IL17-Producing T Cells in Adipose Tissues

This document includes:

-Supplementary figures 1-3

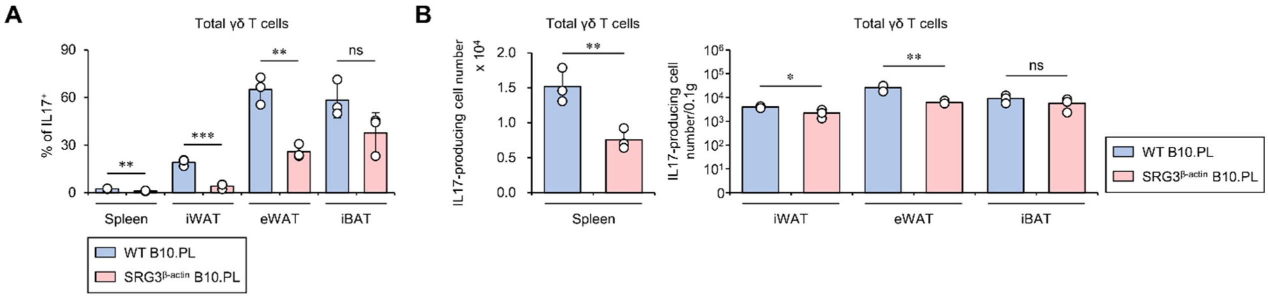

Figure S1. SRG3 overexpression down-regulates the accumulation of IL17-producing γδ T cells in adipose tissues

(A-B) The frequency (A) and cell number (B) of $IL17^{+}$ γδ T cells ($CD45^{+}CD3^{+}$γδ TCR^{+}) from the spleen, iWAT, eWAT, and iBAT were determined by flow cytometry in WT B10.PL and $SRG3^{\beta\text{-actin}}$ B10.PL mice. The mean values ± SD ($n = 3$ in A-B; per group in the experiment; One-way ANOVA; *$p < 0.05$, **$p < 0.01$, and ***$p < 0.001$) are shown. One representative experiment of two experiments is shown. ns, not significant.

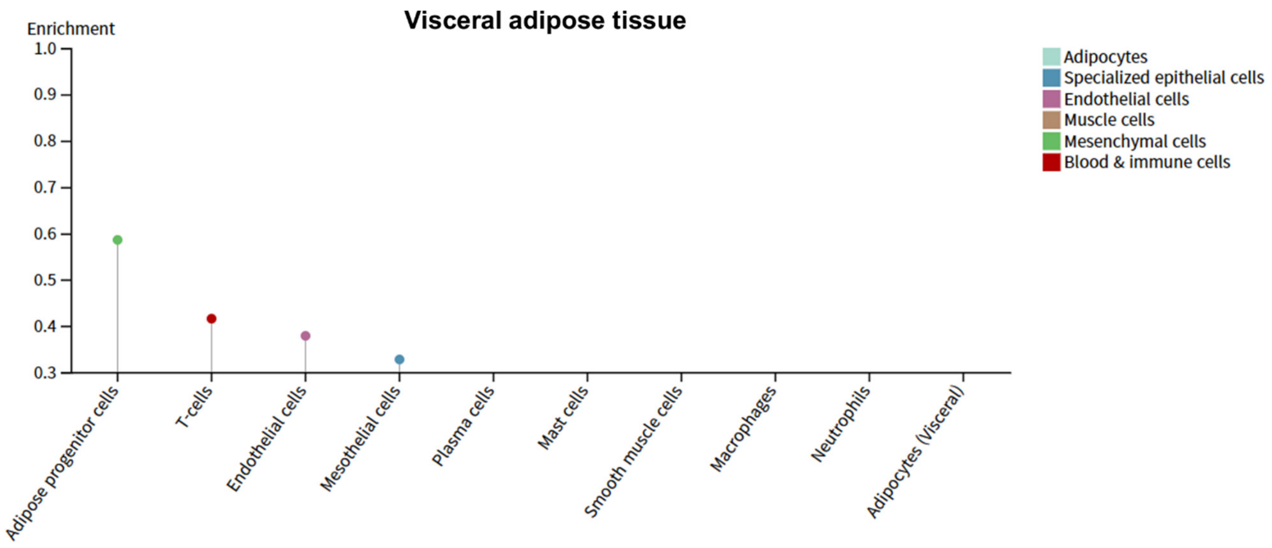

FIGURE S2. Expression pattern of SRG3/SMARCC1 in different cell populations of human visceral adipose tissue.

The mRNA expression profiles of SRG3/SMARCC1 in diverse cell populations of the visceral adipose tissue were derived from the Human Protein Atlas (http://www.proteinatlas.org/). Deconvolution analysis is represented by the plot with enrichment scores for each cell type (cutoff score = 0.3).

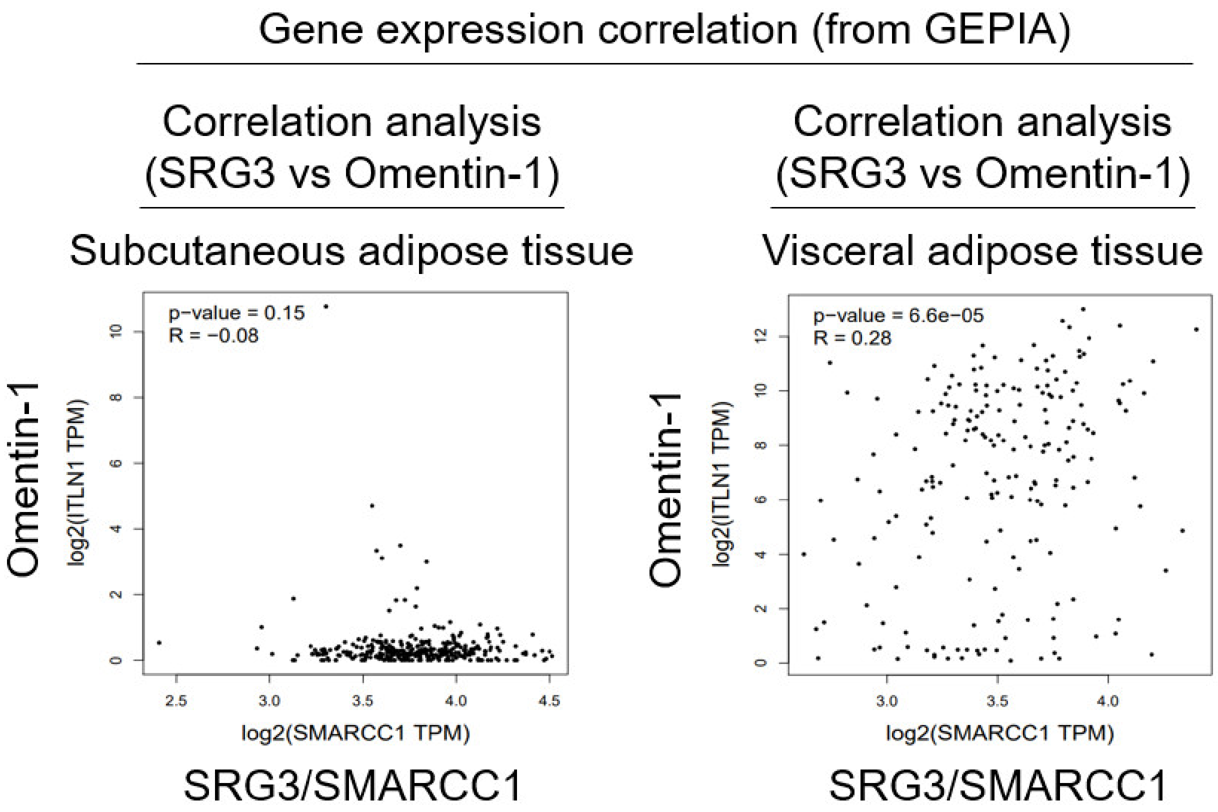

Figure S3. Correlation analyses of SRG3 and omentin-1 gene expression in human adipose tissues.

Pearson correlation analysis of *SRG3* and *ITLN1 (encoding omentin-1)* gene expression was conducted using human subcutaneous and visceral adipose tissue data from the GEPIA (http://gepia.cancer-pku.cn/index.html, accessed on 17 October 2024) tool (TPM; transcripts per million reads).
